# Gender and role models in the education of medical doctors: a qualitative exploration of gendered ways of thinking

**DOI:** 10.5116/ijme.5e08.b95b

**Published:** 2020-01-30

**Authors:** Ola Lindberg

**Affiliations:** 1 Department of Education, Umeå University, Umeå, Sweden

**Keywords:** Role models, gender, medical education, career opportunities, equality

## Abstract

**Objectives:**

To examine how ‘gendered ways of thinking’ relate to role models in medical education.

**Methods:**

This study employed an explorative, qualitative, and cross-sectional design. A total of 57 interviews were held with medical students (28 interviews) and with faculty members (29 interviews) at a Swedish medical school. Participants were asked to describe their role models and the attributes that made certain individuals role models. Data were analysed using an inductive approach in three separate steps that explored the relationship between role models and gender.

**Results:**

Males do not generally consider female doctors as role models, and male role models are generally viewed as more admirable than female role models. This was shown in all steps of the analysis and most prominently in how male role models were described as qualitatively more admirable than female role models. Male role models are thus more common (for male and female students) and described as more admirable. The results point to the persistence of ‘gendered ways of thinking’ that subtly shape medical students.

**Conclusions:**

Gendering role models is disadvantageous to female doctors in several ways, so the results have implications for women’s career paths and opportunities. The results can thus form a basis for discussing and teaching the importance of gender in role modelling and in medical education in general.

## Introduction

The present article investigates how role models in medical education relate to gender. Positive role models are critical in developing professionalism.^1^^-^^3^ Several studies have investigated the attributes of good (and bad) role models.^4^^-^^7^A systematic review of the research on role model attributes concluded that role models are often valued for their patient care, teaching, and personality.^8^ These findings are echoed in a review by Passi and colleagues, who found that excellent clinical, teaching, and personal skills—combined with a humanistic approach—were seen as key attributes of role models.^9^ It has also been convincingly shown that people considered as role models take an active interest in teaching and tutoring.^4^ Previous research also focused on how role-modelling works and how role models can be used in teaching medicine.^10^^-^^12^

Gender and role models have been studied concerning the underrepresentation of women and men in various professions, especially in relation to women’s scarcity in technical professions.^13^ Fields dominated by either gender tend to repel applicants from the underrepresented gender. In medical education, for instance, large surveys have asked whether students believe that female role model would encourage more women doctors to choose surgery.^14^ Jagsi and colleagues studied large cohorts of medical students to find whether same-gender role models influenced students’ choice of speciality.^15^ Where gender inequity has been an issue, people assume that a lack of same-gender role models contributes to underrepresentation.

A surprising lack of studies exists on how role models and role modelling relate to gender. In their 2013 review, Passi and colleagues showed a scarcity of role-model studies addressing culture, diversity, and gender. They recommended this as a direction for future study.^9^ The 2013 review by Jochemsen-van der Leeuw and colleagues did not mention gender as a role modelling issue.8 This lack of studies is more surprising considering how many studies show that male and female experiences of career opportunities, legitimacy as doctors, and expectations as family caretakers differ greatly.^16^^,^^17^ Hill and Giles argue that an ‘illusion of choice’ between specialities exists, given the power that gender has in determining career possibilities.^18^

Besides, those few studies that investigated gender as a factor might have suffered from sampling problems. The influential study by Wright and colleagues on role models’ attributes found no significant gender differences between the group of role models and the control group.^7^ However, the control and role model groups were over 80% male, so the significance test could not have detected a bias for male role models. Magee and Hojat, who explicitly studied male and female role models in medicine, used a sample of pre-selected role models that was 87% male.^19^ In their 2003 qualitative study of excellent role-model characteristics, Wright and Carrese’s sample was 90% men.^6^ In this review, only one study addressed gendered role models with careful sampling and methodology, and it was conducted outside the field of medical education.^20^ It concluded that participants generally (men more than women) would choose same-gender role models.

In sum, although the characteristics of medical role models have been extensively researched, previous research has suffered from certain gender blindness. To date, research on role models has not addressed how gender connects to values and ideals or the ‘natural’, ‘good’, or ‘admirable’. Bleakley urges researchers to go beyond mere demography and biology and, with the help of post-structuralist feminism, focus on problematising gendered ways of thinking and how this affects medical practice and medical education.^21^ Several studies have addressed gender problems in medical education, but there is a longstanding tradition about gender and organisation not yet considered in light of the role model doctor.^17^^,^^22^^-^^24^ This literature shows that gendered ways of thinking profoundly affect views on competence, self-image, and images of others in education and work. Hence, seemingly gender-neutral competency measures (such as clinical skills) tend to depend on ‘performing’ or ‘doing’ gender.^25^^,^^26^ One notable example reports that, in recruitment, female applicants’ credentials are seen by men and by women as less valuable than the same credentials from male applicants.^27^

Starting with the lack of research on role models and gender, this study aimed to examine how ‘gendered ways of thinking’, as described above, relate to role models in medical education. Theoretically, role models are viewed as embodied ideals that signify what is morally good in a certain practice.^28^ Paice and colleagues define role models as ‘people we can identify with, who have qualities we would like to have, and [who] are in positions we would like to reach’.^5^ Such role models indicate the direction and end goal of an educational process: ‘that is who I want to be someday’. In this respect, studying (positive) role models is studying what people regard as morally right or the highest good in a certain practice.

## Methods

### Study design

The study’s design was a qualitative, cross-sectional interview study. A total of 57 interviews with students, teachers, and heads of medical residencies were analysed, using theoretical guidance from a practice-theory perspective where role models can be viewed as embodied ideals.^28^ Thus, embodied ideals reveal the moral dimensions of a ‘generalised medical professional identity’ in undergraduate medical programmes and residencies.^29^

### Setting

The Swedish medical programme is five and a half years long. The programme studied is given by a large university in Sweden in close cooperation with a university hospital. While not a PBL curriculum, the programme relies heavily on clinical cases and on student activity for teaching methods. The programme also entails long clinical placements where students work with professional doctors as tutors, which means students have ample opportunities to observe doctors at work.

### Participants and data collection

The participants were selected for maximal variation from the various groups that shape the culture at a medical school. Thus, students and teachers were important groups in the programme. The heads of medical residencies were also interviewed, as they serve as important gatekeepers to continuing medical training. Participants were selected strategically from lists of students, teachers, and residency heads, and maximal variation in gender and age was pursued. All participants were informed of the study’s aims and that they could terminate their participation at any time. In 2016, the regional ethics board determined that the project did not need ethical approval.

Data were collected on three separate occasions as part of a larger project. Interviews lasted from 30 to 80 minutes and were conducted in Swedish; the author conducted all of them. The interviews included questions about the ‘good medical student’, an excellent (future) doctor’s characteristics, educational methods, and organisational culture. The questions about participants’ personal role models were formulated in the same way on all three occasions. The first round of interviews took place in 2009, including eight interviews with medical students and eight interviews with medical teachers from the same medical school (eight male and eight female). In 2012, the second round of interviews included 21 interviews with heads of medical residencies in various hospitals in Sweden (10 male and 11 female). Finally, the third round in 2017 included 20 interviews with medical students (10 male and 10 female) in their final year at the medical school.

Excerpts of the interviews analysed for this study concerned participants’ personal role models and did not explicitly target or ask about gender. Interviews often provide an ‘idealised moral account’.^31^ While not a disadvantage in a study aimed to describe a practice’s moral structure, participants may still provide accounts that align more with a ‘politically correct’ view than with their own subtle biases. The questions were therefore formulated to avoid mentioning gender as an aspect of role models. Instead, they intended to capture participants’ spontaneous descriptions of their role models. Hence, the three main questions guiding the present analysis were:

        1. Do you have a role model as a doctor?

        2. Can you describe this person to me?

        3. What is it that this person does that you find admirable?

There were no direct questions about the role model’s gender, which was determined by the participant’s use of gendered pronouns or of names identifiable as male or female.

### Data analysis

The total sample comprised 29 men and 28 women (28 students and 29 faculty). Conclusions from these data rely on two main assumptions: (1) that gendered ways of thinking are stable over time and across different specialities as part of a pervasive discourse that permeates society and (2) that education towards a general professional medical identity (including content, methods, and the organisation of clinical versus academic education) is essentially similar throughout Sweden and other countries. The analysis is, therefore, generalizable and likely transferable to similar educational environments.^30^ Interviews were recorded, transcribed verbatim, and analysed in three steps using the qualitative analysis software Nvivo.

In the first step of the analysis, descriptive statistics were used to give an overview of the role models’ genders. The frequencies of having role models, the genders of role models, and the genders of participants were analysed using Microsoft Excel. When a participant discussed several individual role models, only the first-mentioned was included in this step. Descriptions of other role models’ characteristics and attributes, however, were included in the analysis’s following steps. In the second step of the analysis, Nvivo 10’s node structure was used to categorise and juxtapose attributes of role models by gender.^6^ The results are presented in Table 1. The third and final step of the analysis paid special attention to how strongly a person was admired by analysing the participants’ use of superlatives, intensifiers, and other strong adjectives. Thus, role models were categorised according to the strength of admiration expressed in the interviews.

## Results

The results are structured according to the three steps of the analysis described in the Methods section. First, overviews of the identified role models are presented. Second, the role models’ attributes are compared by gender. Third, the results of the analysis of the language used to describe outstanding role models are given.

### Overview of role models

Table 1 shows the frequencies, relative frequencies, and genders of explicitly first-mentioned role models. Only three (5%) of the 57 participants said they did not have a role model. When prompted to describe a role model, 34 (60%) of the role models were identifiable as male or female. Those who did not reveal a gender in their description often had several role models and ‘picked and chose’ from their different attributes. Of the 34 role models with an identifiable gender, 25 (74%) were male, and 9 (26%) were female. Most strikingly, females have both male and female role models (47% and 53% respectively), but males almost exclusively—with only two exceptions—reported that their role models were male rather than female (89% and 11% respectively).

**Table 1:** Frequencies and relative frequencies (in parentheses) of the categories of participants with a gender-identified role model

Categories of participants	Role model male (%)	Role model female (%)
Women	7 (47)	8 (53)
Men	17 (89)	2 (11)
Students	13 (65)	7 (35)
Faculty	11 (79)	3 (21)
Student males	8 (80)	2 (20)
Student females	5 (50)	5 (50)
Faculty males	9 (100)	0 (0)
Faculty females	2 (40)	3 (60)

### Attributes of male and female role models

Comparing female and male role models, respectively, the attributes participants found admirable are presented in Table 2. The number of participants who described each attribute is included to show how commonly a certain quality is attributed to doctor role models.

The three most common role model attributes for both male and female role models were ‘good with patients’, ‘knowledgeable clinician’, and ‘good as a tutor/teacher’. These three categories are also reported as important in several earlier studies.^4^^,^^8^^,^^9^ In the analysis of gender, more attributes specifically describe male role models than female role models. This might be an effect of numbers; 25 male and only 9 female role models were in the sample. However, examining the gender-specific attributes offers evidence that men are allowed more complex personalities as role models. Notably, Table 2 comprises attributes of the whole material; it is not a selection. Thus, there are simply fewer characteristics that describe female role models. For instance, male-specific attributes include ‘charisma’, ‘educated (non-medical)’,

**Table 2:** Attributes of male (n = 25) and female (n = 9) role models

Attributes used to describe both male and female RMs	M/F
Good with patients	(10/6)
Knowledgeable clinician	(8/7)
Good as a tutor/teacher	(8/4)
Able to create an atmosphere of trust	(5/2)
Able to listen	(4/2)
Humble	(4/2)
Straightforward	(4/1)
Conscientious	(3/1)
Empathetic	(3/1)
Likeable personality	(2/2)
Strong integrity	(2/2)
Passionate	(2/1)
Attributes used only for male RMs	Attributes used only for female RMs
Ability to handle problematic situations (3)	Showed confidence in me (2)
Availability (3)	Competent *and* a woman (1)
Good sense of humour (3)	Competent despite family issues (1)
Ability to command respect (2)	
Consideration of others (2)	
Curiosity (2)	
Sense of duty (2)	
Leadership skills (2)	
Patience (2)	
Charisma (1)	
Education (non-medical) (1)	
Ethical pathos (1)	
Intelligence (1)	

‘humorous’, and ‘ethical pathos’. These are favourable personal characteristics, but they are more generic and more peripherally related to clinical work than those used to describe female role models. The few described female role model attributes mostly relate to their professional function, and all are reported only by female participants who also indicate the importance of their role model’s gender. In one case, a woman is considered a role model simply because she is a woman:

“I like when there is a lecturer who is a woman and very good at what she does… I know more girls in the group who think so, too. That we look up to her instead of all these 50-year-old men.” (Female, student)

In another instance, a woman is considered a role model because she handles personal hardships while still performing well at work:

“[She] has been very good at combining high-end research with clinical work, and, since I happen to know this, a very troublesome family situation.” (Female, faculty)

### Strength of admiration

The most striking differences between the accounts of male and female role models are not in the attributes but in the adjectives and intensifiers used to describe them. Words signalling the degree of admiration (for example, ‘Is the role model’s communication with patients good, excellent, or outstanding?) differed for male and female role models. Descriptions of role models yielded nine ‘outstanding’ role models and 25 ‘normal’ role models, and ‘outstanding’ role model behaviour was ascribed to eight male role models but only one female. This is the whole set of quotes in this category—not a selection. In this sample, only one quote described an ‘outstanding’ female role model. Table 3 shows descriptions of men and women in the ‘outstanding’ category.

Male and female role models are described very differently. Only males are described with adjectives like ‘magical’, ‘genius’, or ‘fantastic’. Notably, these descriptions come from both male and female participants.

**Table 3:** Descriptions of the nine ‘outstanding’ male (n = 8) and female (n = 1) role models

Description of a male role model	Description of a female role model
‘It’s when he meets a patient… He is a social genius’ (female, student)	‘She had a really good relationship to patients. It was almost as if patients hugged her at the end and thanked her.’ (female, student)
‘He is like… well he is *magical*. I felt that I could ask anything without feeling stupid’ (female, student)	
‘He was so incredibly good, like, he knew *everything*’ (male, student)	
‘If I could become half the doctor he is, I would be really successful’ (female, student)	
‘He was very, very, *very* knowledgeable in his area too’ (male, faculty)	
‘A completely, thoroughly good person’ (male, student)	
‘A fantastic person, a good person’ (female, faculty)	

## Discussion

This study aimed to explore the relationship between role models and gender in medical schools, and three main findings were identified. First, males do not generally have female role models, but women have both male and female role models. Second, the most commonly admired attributes are similar in both male and female role models. However, male role models are admired more for personal attributes not directly related to work than are female role models. Male role models are more likely to be admired for their personalities than for their professionalism. Finally, male role models are generally described qualitatively as more admirable than their female counterparts.

The results show a clear gendered difference in how students and faculty view male and female doctors. Portraying this as a problem reasonably assumes there are no real gendered differences between how men and women perform in medical practice: men and women are not inherently different in aptitude or suitability for outstanding medical professionalism. Interestingly, the students were exposed to a workforce of teachers and clinicians who were 51% female in a country with an international reputation for gender equality and with consistent top ranks in gender equality indexes.^32^^,^^33^ Thus, no reasonable explanation exists for this study’s results other than a subtle and unconscious gender bias not apparent in demographic statistics.

That men almost exclusively have male role models should not be entirely surprising. Earlier research on gender and role models reported this, but it was rather hidden in those papers. Previous studies^6^^,^^7^^,^^19^ used samples of pre-chosen role models identified by students or faculty representatives. That these samples of role models were almost all male (>80%) should perhaps have been reported as an anomaly or provoked interest in why the samples were so skewed.

Several previous studies have shown the importance of role models in developing professionalism.^1–3^ If role modelling is gendered, as the results of the present study suggest, severe implications exist on what kind of professionalism this process develops. If men do not consider women as role models, for example, they exclude half of the models of good medical of practice available to them. Furthermore, as gendered role models are disadvantageous to female doctors in several ways, the results also have implications for women’s career paths and opportunities. When and how, for instance, is it relevant to speak of the feminisation of the medical profession if the admirable doctor is still male?^34^ When considered for promotion or for a new position, how is a female doctor’s competence viewed and communicated differently from that of a male doctor? This kind of subtle and unconscious bias might also help explain the ‘glass ceiling’.^35^

### Limitations

This article explores an area clearly lacking in research. The conclusions and main contribution of this article therefore contribute to a plausible hypothesis about gender assumptions in role modelling that merits further study in other settings and via different methodologies. A limitation of the present study is, naturally, the sample size, which does not adequately convey how skewed is the ratio of female/male role models. Another limitation is that one researcher conducted the coding and categorisation, so there are reasons to be careful with the conclusions based on qualitative categories. Finally, sampling from a single medical school and rather crude categories (students and faculty) limit the present study.

### Suggestions for further research

While having some limitations, the present study opens up interesting new ways to research role models. Role models as a gendered concept should be further investigated, and this study could inform such attempts. First, large scale surveys could investigate the conjecture that men have no female role models by using samples representative of a larger population of students and faculty members. Second, the study shows that comparing modelled attributes or behaviours tells only part of the story. Roughly similar role model characteristics are described for both male and female role models (consistent with earlier research), but the wording and strength of these descriptions are very different. Future studies should therefore also consider wording and strength of admiration to analyse whether gendered differences exist among who becomes a role model and why. This could pertain to qualitative and quantitative approaches.

## Conclusions

This exploratory study’s results suggest that the gendering of role models is a result of the pervasive ‘gendered ways of thinking’ that permeate medical practice and education.^21^ Considering the implications for medical education, this study shows that the case for gender equality reaches beyond a gender-balanced workforce. Students and teachers should be consciously aware of the subtle biases that cause men to be more admired than women as professionals, perhaps by using studies such as this as a basis for discussions in professionalism courses. Who we choose as role models affect our general view of who doctors should aspire to be. If that doctor is generally male, and if males are admired more than women, there is a constant, subtle, and often unconscious depreciation of women’s efforts to practise medicine.

### Conflict of Interest

The author declares that he has no conflicts of interest.
